# 

**Published:** 1844-09

**Authors:** 


### AMERICAN JOURNAL

OF

### Dental Science.

PUBLISHED UNDER THE AUSPICES OF THE

### American Society of Dental Surgeons.

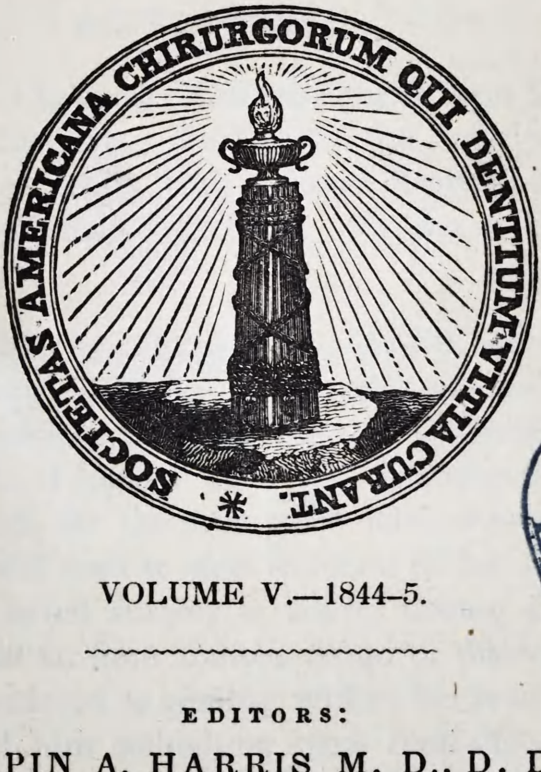

VOLUME V.—1844-5.

EDITORS:

CHAPIN A. HARRIS M. D., D. D. S.
EDWARD MAYNARD, M. D., D. D. S.
AMOS WESTCOTT, M. D. D. D. S.

SURGEON GEN'L'S OFFICE LIBRARY. 13373
Surgeon Genl's Office LIBRARY. 13373 Washington, D.C.

BALTIMORE:
ARMSTRONG & BERRY.
WOODS AND CRANE, PRS., BALT.

1844.

*To Subscribers.*—We hope that all who are in arrears for the Journal, will immediately on the reception of this number, forward the amount of their indebtedness. The terms are payment in advance, and if all our subscribers would comply with this requisition, the conductors would be saved a vast amount of labor and trouble, to which they are now subjected, in meeting the expenses of the publication.

*To Correspondents.*—We have received articles for publication, which we are under the necessity of reserving for the next number.

*Gossip with our Profession.*—The directions for plugging teeth, given by some writers upon dental surgery, are really very amusing. For instance, one author, after directing the operator to make a *rope* of the foil he is to use, says—"the cavity being clean and dry, one end of this little rope is placed at the bottom of it, and by a small instrument it is folded over and over, and round and round, until the cavity is full!" &c. &c. Now, this may be what some English writers call "*stuffing*" a tooth, indeed we think it must be, for not only the directions but the result of the operation will be found, in practice, "all stuff." One may be *shown* how to plug teeth, as one may be shown how to paint a picture; but one has never yet been *told* how to do either. . . . . Cuyler & Sevords, hardware merchants, New York, have for sale the famous *Arkansas oil stone,* so highly prized by all dentists who have had the very great luck to obtain a piece of it. All surgeons, general or dental, should have a piece of this stone put up with their instruments. To say that it is "worth its weight in gold" is to say less than the truth; there can be no more comparison between the worth of this stone and gold than there can be between the worth of a skilful dentist's services and the price he gets for them. . . . . We would respectfully suggest to all dentists who undertake to teach students, that they have copies of all the varieties of human teeth made of *clear glass*, with cavities cut in them to represent the cavities prepared for plugging in the natural teeth. If the surfaces of the teeth and cavities be cut smooth, the student will be able to see the bottom and sides, as clearly as he can the top, of the plug he inserts. Mr. Simpson, lapidary seal-engraver, Baltimore, an accomplished artist, would, we doubt not, imitate perfectly, in glass or crystal, the shape of any tooth left with him. . . . . Chevalier, (see his advertisement) makes an improved punching forceps for plate work. By a very simple, and therefore the more valuable, contrivance, the punch is withdrawn from the plate in which it makes a hole, by the act of opening the blades. The advantage to be gained will be perceived at once by all who have used the tools this is to supercede. As to the dental instruments made by Chevalier, Arnold and others, we have heard them spoken of in very high terms, but cannot judge of their value from the use of them, nor indeed of those of *any other* cutler, having never used any dental instruments (except forceps, and these we remodel, and files) but those made by *ourself.* With deference to the opinions of others, we think all dentists should make their own excavating, plugging and scaling instruments. We will even go so far as to say that—entertaining our present views of the qualifications necessary to practice dental surgery properly, and of our duty to our patients—if we *could not* make those instruments we should do both our profession and the public an act of justice by changing our occupation. It requires a far inferior grade of mechanical talent and skill to make any implement we use than to do *justice* in a difficult operation upon the teeth. A most happy and just remark was made by the president of the American Society of Dental Surgeons—that "the *hands* of the dentist should be educated as well as the *head;*" and this manual education can only be obtained by long use—use from boyhood to manhood—of small tools;—large or heavy ones will not only make the hands too large, but they destroy that pliability and delicate sense of touch, which, if not so necessary for the success of the operation, is all important for the feelings and welfare of the patient. We are frequently applied to for instructions in our profession, and the first question we ask the applicants is, "have you been accustomed to any labor requiring some mechanical skill, and the use of the hands?"—if not, the man is dismissed as not prepared to *commence* his studies—advised, perhaps, to turn his attention to law, or some other profession that requires the head *only* to be educated, and answered that for the profession of dental surgery, his preparatory course should have commenced as early as his tenth year.

M.

*Washington, August,* 1844.

### EDITORS' PROSPECTUS

OF THE

### American Journal and Library of Dental Science.

As this Journal has become the organ of the American Society of Dental Surgeons, and as its name has been somewhat modified, it may be considered as having entered upon a new period of its existence; and its former publishers congratulate themselves and the profession on its good fortune. Its perpetuity is now considered as settled. The profession will always need a periodical devoted to its interests, and who is better able to conduct it, than the only regularly organized and extensive association of dental surgeons in the world. We repeat, that we congratulate ourselves and our professional brethren on an event so auspicious to the science of dental surgery, as the formation of a national society and the transfer of the journal to that body. The work can now be regularly issued, whether its circulation be co-extensive with the profession or not. If the subscription should be limited, the issues can be made to correspond with the demand, so that the expense to the society may be kept wholly within its means.

The American Journal and Library of Dental Science, is now proposed to take an independent ground, and will hereafter solicit no favours from those members of the profession who are opposed to the dissemination of periodical information. To others who are favourably inclined to a community of knowledge, this journal will not fail to commend itself, as one of the most efficacious means of exposing the impositions of quackery, and imparting to honorable pretensions that kind of information which their usefulness and talents should secure.

The journal will be regularly issued on the first of December, March, June and September, annually, at the enhanced price of five dollars per volume, payable in all cases in advance. The price of subscription may be paid to the treasurer of the society, Dr. C. A. Harris, Baltimore city, or to either of the editors, or any of the publishers, collaborators or acknowledged agents of the journal. Advance payment is required by a special resolution, passed at the last meeting of the society, and is necessary to protect it from pecuniary loss, a neglect of which has involved the publishers of the first volume in unexpected embarrassment, if not in ultimate loss.

To save postage, the agents in all the large cities of the United States, and Great Britain, will be furnished with copies to supply subscribers, by such conveyance as they shall prescribe. And where it can be done, even individuals may receive their numbers by private conveyance if they choose.

With these few explanatory remarks, we commit our work to its coming destiny, after assuring the profession that no efforts will be spared to render it every way worthy of the great subject to which its pages will be exclusively devoted.

CHAPIN A. HARRIS,
EDWARD MAYNARD,
AMOS WESTCOTT.

---

Besides the Collaborators, the following gentlemen are regularly authorized

### AGENTS OF THIS JOURNAL.

Bradbury & Soden,.........Boston.
John M'Connell, M. D.....Richmond, Va.
O. P. Laird, M. D.........Columbus, Ga.
Geo. Washington Parmly, New Orleans.
G. McDonald, M. D .......Macon, Ga.
Blandin & Reynolds,.....Columbia, S. C.
Eleazar Gidney, D. D. S...Manchester, Eng.
Drs. Goddard and Ferguson,Louisville, Ky.
James Taylor, M. D.. ....Cincinnati, Ohio.
Nicholas Clute,...........Louisville, Ky.
J. N. Frink,........... ....Portland, Me.
E. J. Dunning,.............Buffalo, N. Y.
Benj. Strickland,.........Cleveland, Ohio.
B. B. Brown, M. D.........St. Louis, Mo.
W. R. Scott, D. D. S........Raleigh, N. C.
S. M. Shepherd,............Petersburg, Va.
W. W. H. Thackston, D. D. S. Farmville, Va.
Edward Taylor, M. D.. ...Natchez, Miss.

